# Physicochemical and Antifungal Properties of Clotrimazole in Combination with High-Molecular Weight Chitosan as a Multifunctional Excipient

**DOI:** 10.3390/md18120591

**Published:** 2020-11-26

**Authors:** Bożena Grimling, Bożena Karolewicz, Urszula Nawrot, Katarzyna Włodarczyk, Agata Górniak

**Affiliations:** 1 Department of Drug Form Technology, Wroclaw Medical University, Borowska 211 A, 50-556 Wroclaw, Poland; bozena.grimling@umed.wroc.pl; 2 Department of Pharmaceutical Microbiology and Parasitology, Wroclaw Medical University, Borowska 211 A, 50-556 Wroclaw, Poland; urszula.nawrot@umed.wroc.pl (U.N.); katarzyna.wlodarczyk@umed.wroc.pl (K.W.); 3 Laboratory of Elemental Analysis and Structural Research, Wroclaw Medical University, Borowska 211 A, 50-556 Wroclaw, Poland; agata.gorniak@umed.wroc.pl

**Keywords:** clotrimazole, chitosan, drug–polymer combination, synergistic antifungal activity, *Candida* spp.

## Abstract

Chitosans represent a group of multifunctional drug excipients. Here, we aimed to estimate the impact of high-molecular weight chitosan on the physicochemical properties of clotrimazole–chitosan solid mixtures (CL–CH), prepared by grinding and kneading methods. We characterised these formulas by infrared spectroscopy, differential scanning calorimetry, and powder X-ray diffractometry, and performed in vitro clotrimazole dissolution tests. Additionally, we examined the antifungal activity of clotrimazole–chitosan mixtures against clinical *Candida* isolates under neutral and acid conditions. The synergistic effect of clotrimazole and chitosan S combinations was observed in tests carried out at pH 4 on *Candida glabrata* strains. The inhibition of *C. glabrata* growth reached at least 90%, regardless of the drug/excipient weight ratio, and even at half of the minimal inhibitory concentrations of clotrimazole. Our results demonstrate that clotrimazole and high-molecular weight chitosan could be an effective combination in a topical antifungal formulation, as chitosan acts synergistically with clotrimazole against non-albicans candida strains.

## 1. Introduction

Excipients are inactive ingredients that perform defined functions in pharmaceutical preparations. Examples include bulking, solubilising, hydrophilising, gelling, and viscosity-increasing agents. Advances in drug formulations and delivery systems have led to the development of multifunctional excipients, agents which possess additional functions aside from their primary purpose. These multifunctional excipients can favourably influence the properties of drugs [1,2]. Macromolecular compounds, such as chitosans, belong to this group of excipients. Chitosans can be used as disintegrants, film-forming agents, coating agents, tablet binders, viscosity enhancers, and mucoadhesive agents [3,4]. Chitosans also have a broad spectrum of biological properties that have been well described in the scientific literature, including antibacterial, antioxidant, haemostatic, and antifungal activity [5,6,7]. Many of chitosan’s physicochemical and biological properties, including its antifungal activity, are related to its molecular weight (MW) and degree of deacetylation [8,9,10,11]. Low-molecular weight chitosan (LMWC), i.e., less than 150 kDa, is more successful in inhibiting mycelial growth than high-molecular weight chitosan (HMWC). This difference is a consequence of its enhanced interactions with cell membranes, allowing the compound to successfully disturb membrane function and inhibit major metabolic processes after penetrating the fungal cell [12]. Calamari et al. evaluated the effect of HMWC and sodium alginate on *Candida albicans* proteinase activity. Their results indicated that HMWC (300 kDa) chitosan significantly inhibits the proteinase secretion of *Candida albicans* [13].

*Candida* species are the predominant cause of fungal infections, and *Candida albicans* is the most commonly identified opportunistic pathogen of the *Candida* genus in clinical contexts. In recent years, *Candida glabrata* has become the second most common cause of mucosal and invasive candidiasis, responsible for 15–20% of all known *Candida* infections [14]. Notably, the incidence of *Candida* infections increases every year [15].

The emergence of antifungal resistance has created the need for new formulations that can effectively treat candidiasis [12]. There are several classes of drugs used as fungal therapies, and azole derivatives are most frequently employed to treat *Candida* infections [16]. One example is clotrimazole, a synthetic imidazole derivative with broad-spectrum antifungal activity [17]. The low aqueous solubility (0.49 mg/L) of this active pharmaceutical ingredient (API) is an obstacle to achieving sufficient concentrations for topical application of the drug [18,19]. To prepare clotrimazole suspensions with potent antimycotic activity, excipients that improve solubility and the dissolution rate must be incorporated [20,21,22,23,24,25]. 

The wide spectrum of chitosan biopharmaceutical properties, including the ability for fungal growth inhibition, prompted us to examine chitosan’s potential as a multifunctional carrier in a solid mixture with the most employed antifungal API. For the first time, we investigated the possibility of combining chitosan and clotrimazole in simple powder solid mixtures. In this study, we posited that a formula containing clotrimazole and HMWC as an excipient could act as an effective antifungal agent. Our work aimed to determine the physicochemical properties of solid powder formulations. We carried out in vitro dissolution studies and determined the antifungal activity of solid clotrimazole mixtures (prepared by grinding or kneading) with three samples of high-molecular weight chitosan: chitosan A, chitosan B, and chitosan S.

## 2. Results and Discussion

### 2.1. Clotrimazole Content

We first determined the clotrimazole content in mixtures obtained by grinding (GM) and kneading (KM) methods (Table 1). We determined that the clotrimazole content ranged between 96.52% and 101.83%. 

### 2.2. Spectroscopic Studies of Clotrimazole–Chitosan Mixtures

**Figure 1:**
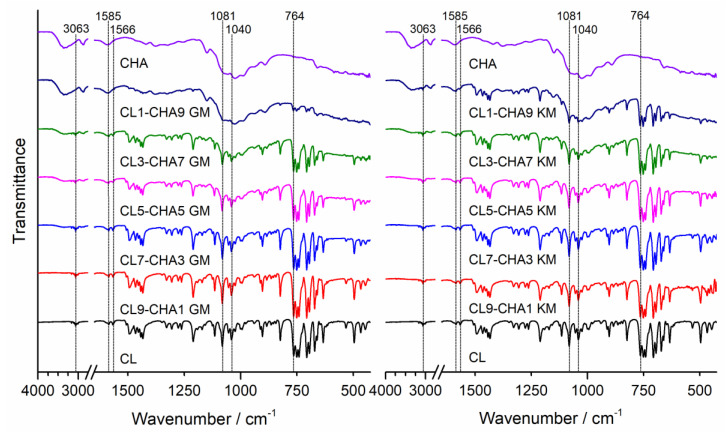
FTIR spectra of clotrimazole (CL), chitosan A (CHA), and CL–CHA mixtures prepared by grinding (GM) and kneading (KM) methods.

**Figure 2:**
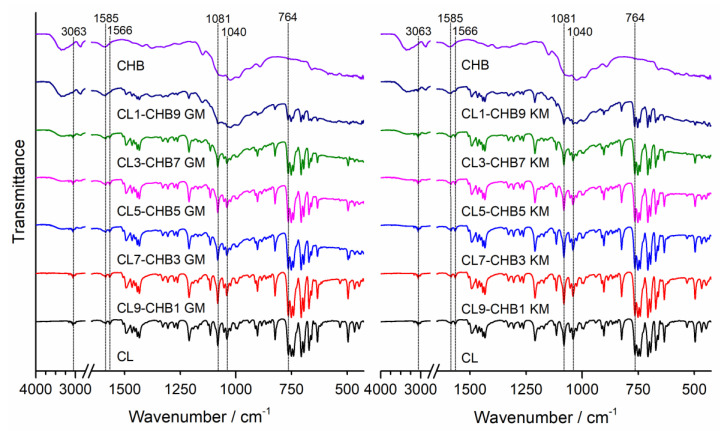
FTIR spectra of clotrimazole (CL), chitosan B (CHB), and CL–CHB mixtures prepared by grinding (GM) and kneading (KM) methods.

**Figure 3:**
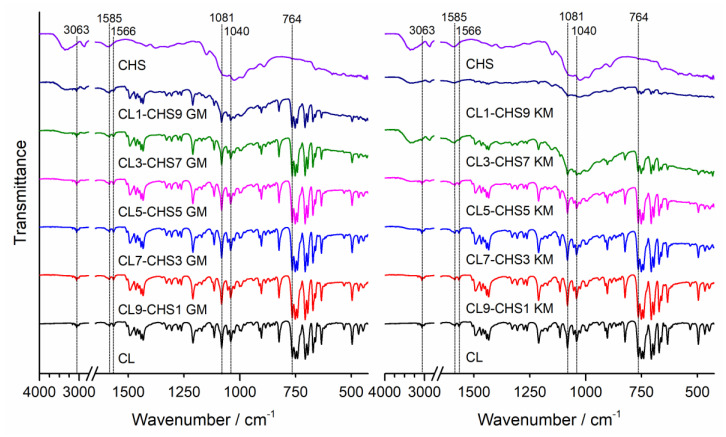
FTIR spectra of clotrimazole (CL), chitosan S (CHS), and CL–CHS mixtures prepared by grinding (GM) and kneading (KM) methods.

To determine possible interactions in the obtained solid clotrimazole and chitosan mixtures, we obtained Fourier-transform infrared spectroscopy (FTIR) spectra and powder X-ray diffraction (PXRD) patterns. FTIR spectra for clotrimazole (CL), chitosan, and their corresponding mixtures are shown for CHA, CHB, and CHS in Figure 1, Figure 2 and Figure 3, respectively. The characteristic absorption bands observed on clotrimazole FTIR spectra, corresponding to aromatic C-H stretching (3063 cm^−1^), aromatic C=C stretching (1585 cm^−1^), C=N stretching (1566 cm^−1^), C-N stretching (1081 cm^−1^, 1040 cm^−1^), and aromatic C-H bending (764 cm^−1^), are consistent with the literature data [26]. The FTIR spectra obtained for different chitosan types (CHA, CHB, and CHS) showed wide absorption bands in the 3280–3360 cm^−1^ region, related to the superposition of O-H and N-H stretching, which is characteristic for polysaccharide compounds. Absorption bands were also observed on all registered chitosan spectra in the region of 2870–2930 cm^−1^, corresponding to the presence of C-H aliphatic stretching vibrations, at 1024 cm^−1^, attributed to the C-O stretching, and at 1150 cm^−1^, attributed to the asymmetric stretching of the C-O-C bridge (typical for glycoside linkages) [27]. The FTIR spectra registered for all prepared CL–CH mixtures showed a characteristic intensity reduction compared to the bands corresponding to the pure drug. No additional bands were observed in the registered spectra that could indicate chemical interactions between the drug and chitosan in the prepared mixtures, as well as no shifts or broadening of bands that could indicate drug–polymer hydrogen bonds.

**Figure 4:**
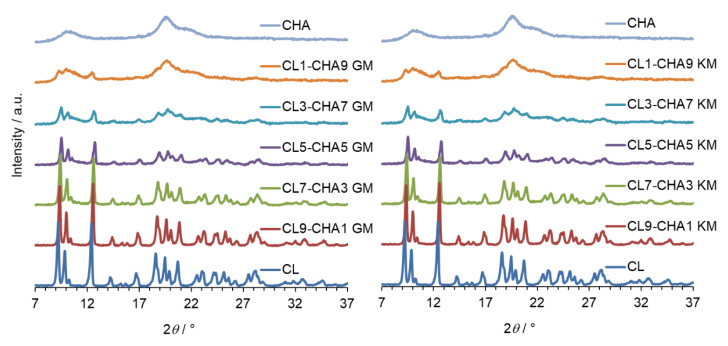
PXRD patterns of clotrimazole (CL), chitosan A (CHA), and CL–CHA mixtures prepared by grinding (GM) and kneading (KM) methods.

**Figure 5:**
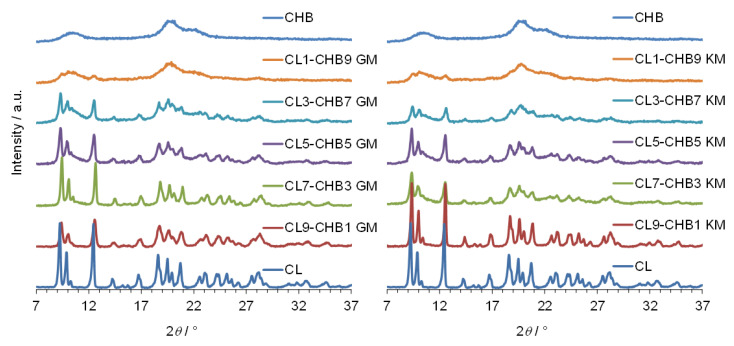
PXRD diffraction patterns of clotrimazole (CL), chitosan B (CHB), and CL–CHB mixtures prepared by grinding (GM) and kneading (KM) methods.

**Figure 6:**
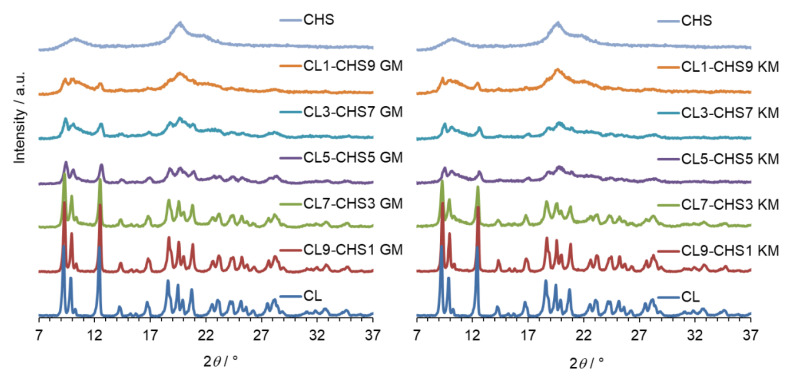
PXRD patterns of clotrimazole (CL), chitosan S (CHS), and CL–CHS mixtures prepared by grinding (GM) and kneading (KM) methods.

The same conclusion can be drawn from analysing PXRD observations, shown in Figure 4, Figure 5 and Figure 6 for CL–CHA, CL–CHB, and CL–CHS mixtures, respectively. According to published data [26], the CL diffractogram shows distinct peaks successively at 2*θ*: 9.3°, 10.0°, 12.5°, 16.8°, 18.7°, 20.8°, 22.6°, 24.3°, 27.7°, and 28.2°. These PXRD data are compatible with the structure of clotrimazole presented in the Cambridge Structural Database (CSD ref. PUTRIH) [28]. Consistent with Yen at al. [29], the broad peaks at an angle 2*θ* of 10° and 20° occurred for all analysed chitosans and examined mixtures; these peaks correspond to the crystalline phase dispersed in the more hydrated and amorphous phase [30]. The comparative analysis of diffraction patterns obtained for the CL–CH mixtures shows a decrease in CL diffraction intensity for each of the mixtures obtained by grinding and kneading methods, as a consequence of the reduction in the CL content in the mixture, crystallinity decrease, and an effective dispersibility in the polymer matrix [31].

### 2.3. In Vitro Dissolution Studies

**Figure 7:**
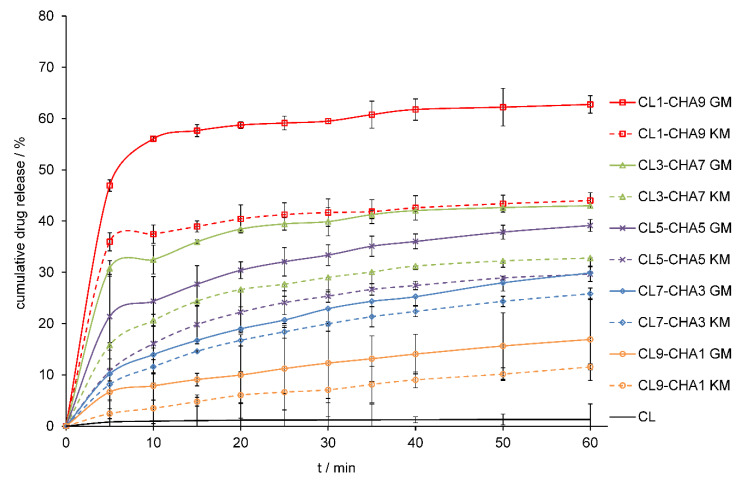
Dissolution profiles of CL and CL–CHA mixtures obtained by grinding (GM) and kneading (KM) methods in 1.0% SLS at 37 °C. Results are expressed as mean ± S.D. (*n* = 3).

**Figure 8:**
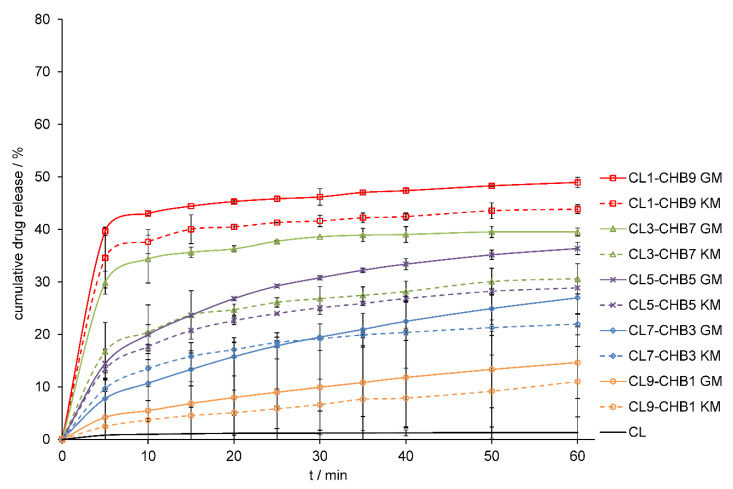
Dissolution profiles of CL and CL–CHB mixtures obtained by grinding (GM) and kneading (KM) methods in 1.0% SLS at 37 °C. Results are expressed as mean ± S.D. (*n* = 3).

**Figure 9:**
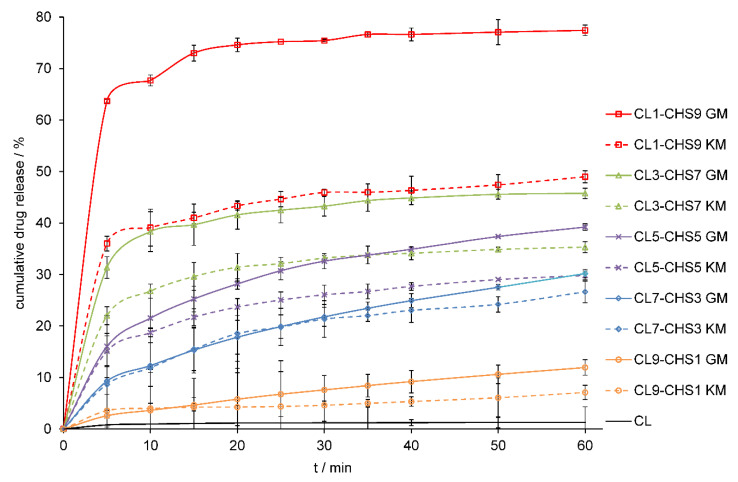
Dissolution profiles of CL and CL–CHS mixtures obtained by grinding (GM) and kneading (KM) methods in 1.0% SLS at 37 °C. Results are expressed as mean ± S.D. (*n* = 3).

The method of preparing the solid-state formulation influences on its physicochemical properties, such as the dissolution rate of API from a solid drug–polymer mixture. In the study, two different methods of preparing clotrimazole–chitosan mixtures: the solvent-free method of mechanochemical grinding and the method with the use of a solvent, were selected. The grinding method appears as a fast, highly efficient, convenient, and eco-friendly solvent-free solid mixtures formulation method, and the second method used, the kneading method, can be used as an industrially prepared solid binary system. Numerous reports indicate that chitosan can improve the dissolution rate and bioavailability of drugs with poor solubility [31,32,33,34,35,36,37,38]. Therefore, we compared the dissolution profile of pure CL in 1.0% sodium lauryl sulphate (SLS) to the dissolution profile of CL released from CL–CHA, CL–CHB, and CL–CHS mixtures obtained from different drug/polymer weight ratios. Figure 7, Figure 8 and Figure 9 present CL dissolution profiles following release from CL–CHA, CL–CHB, CL–CHS mixtures, respectively. The addition of chitosan causes the CL dissolution rate to increase, independent of the chitosan molecular weight, even in the first 5 min of the dissolution test. The CL dissolution rate also increases with enhanced chitosan content in the appropriate CL–CH mixture. All CL–CH mixtures containing 90 wt.% polymer had the best CL dissolution profile, an observation also made for naproxen–chitosan [31] and telmisartan–chitosan [32] solid systems. We found that the grinding method was more effective than the kneading method, which is also more difficult to scale-up for industrial applications, as it requires the addition of an organic solvent and using high temperatures [33]. The dissolution highest rate was observed for mixtures obtained by grinding with chitosan S, which has the highest molecular weight. An almost 60-fold increase in CL cumulative release was obtained for the CL1–CHS9 GM mixture compared to the pure drug dissolution profile after 60 min (Table 2). The effectiveness of chitosan on antifungal drug release from various dosage forms was confirmed by Giunchedi et al. [39] in in vitro and in vivo studies. Additionally, combination therapy can result in a synergistic effect that leads to drug dose reduction, limiting the development of antifungal resistance [40].

### 2.4. Differential Scanning Calorimetry (DSC) Analysis

**Figure 10:**
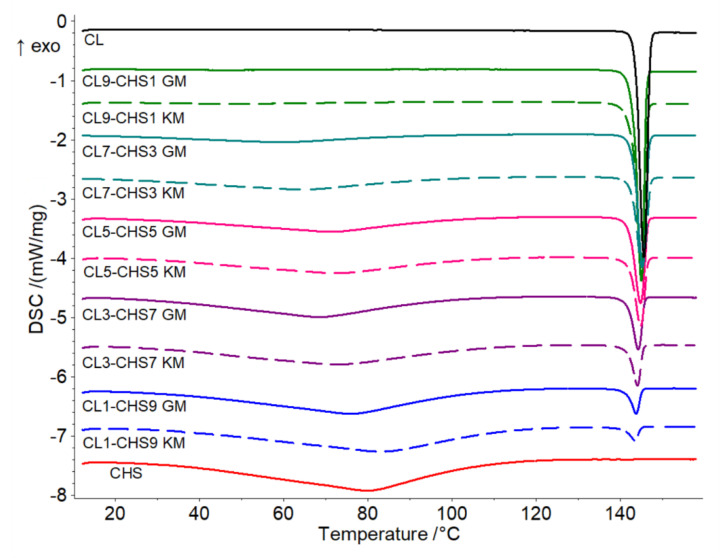
DSC thermograms of clotrimazole (CL), chitosan S (CHS), and CL–CHS mixtures prepared by grinding (GM) and kneading methods (KM).

Differential scanning calorimetry (DSC) thermograms registered for CL–CHS mixtures confirm the thermal stability of the drug. The heating curve of pure clotrimazole (Figure 10) shows a sharp, single endothermic peak at 145.6 (∆H = 103.5 J g^−1^) related to its melting, whereas the thermogram for chitosan S shows a broad endothermic event in the range of 40–110°, indicating an evaporation of residual moisture present in the polymer sample [41]. Table 3 contains the temperatures and enthalpy values of endothermic events related to the melting of CL, visible on DSC curves registered for CL–CHS mixtures. An increase in chitosan concentration causes a reduction in CL melting enthalpy. The sharp shape of the clotrimazole melting peak, visible on DSC curves of all examined mixtures, indicates that the substance remains in a crystalline form. Only slight shifts in the clotrimazole melting peak towards lower temperatures were observed on the DSC curves. The same trends were observed for mixtures obtained by kneading. No decomposition was observed in any tested mixtures after heating to 160 °C. Finally, there were no new thermal effects, which could indicate the occurrence of drug–polymer interactions.

### 2.5. Analysis of Antifungal Activity

Based on their superior dissolution properties, we examined the anticandidal activity of clotrimazole–chitosan S combinations, under neutral and acid conditions. The minimal inhibitory concentrations of chitosan S (MIC_CH_), determined in neutral pH 7 (Table 4), indicated that it had no activity against tested representatives of *Candida glabrata* and *Candida albicans* (M.I.C. > 250 mg/L). The statistical analysis was performed by Student’s t-test, and *p* values of <0.05 were considered as significant. 

Following the addition of 0.1% acetic acid to the growth medium, the MIC_CH_ decreased for all tested strains as well as for four clinical isolates (three strains of *C. glabrata* and one strain of *C. albicans*) to 0.8–3.9 mg/L. In the case of 13 isolates (9 strains of *C. albicans* and 4 strains of *C. glabrata*), the values of MIC_CH_ did not change at pH 4. 

The MIC of clotrimazole (MIC_CL_), determined in standard conditions (pH 7), ranged from 0.125 to 4 mg/L for *C. glabrata* and from <0.0156 to 2 mg/L for *C. albicans*. In acidic conditions (pH 4), the MIC of clotrimazole increased from 8- to 30-fold; the only exceptions were two isolates of *C. albicans* (No 1050 and 1342), which showed higher MIC_CL_ at a standard dose (2 mg/L), and decreased to 0.03 and 0.0156 mg/L, respectively, at pH 4. The antifungal activity of clotrimazole in combination with chitosan S was independently tested in four strains—*C. glabrata* 769, *C. glabrata* 773, *C. glabrata* 1941, and *C. albicans* 488—in standard and modified conditions. The chequerboard assay indicated that chitosan and clotrimazole were synergistic at an acidic pH against *C. glabrata* isolates (FICI of 0.072, 0.0796, and 0.2644). In the case of *Candida albicans* 488, indifference was observed, independent of pH (Table 5 and Table 6).

**Figure 11:**
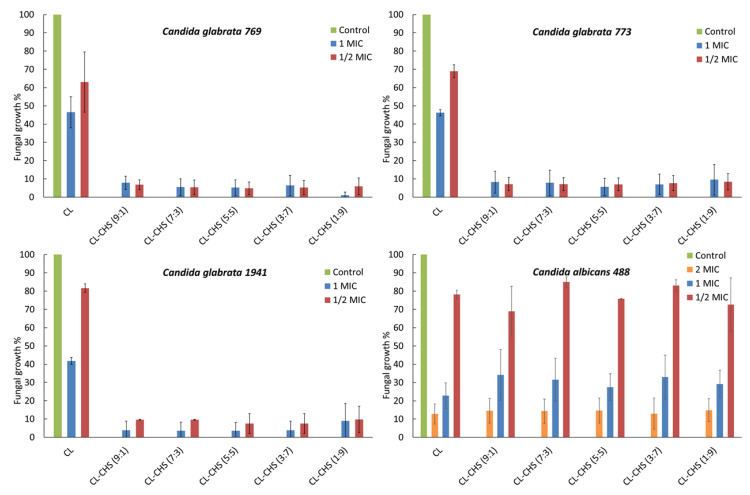
Fungal growth in the presence of clotrimazole (CL) and chitosan S (CHS) at pH 4. Minimal inhibitory concentration (MIC) = minimal inhibitory concentration of clotrimazole for tested strain, ½ (MIC) = clotrimazole concentration equal to half of the value of MIC, 2 MIC = clotrimazole concentration equal to twice the value of MIC, CL–CHS = mixtures of clotrimazole with chitosan S at the weight ratios of 1:9, 3:7, 5:5, 7:3, and 9:1.

These findings were supported by tests of antifungal activity at different weight ratios of CL–CH mixtures. The antifungal activity of clotrimazole and chitosan mixtures at pH 4 is presented as the percentage of fungal growth compared to the control (Figure 11). In all examined *Candida glabrata* strains, the inhibition of fungal growth reached at least 80%, regardless of CL–CH weight ratio. On the other hand, the addition of chitosan showed no substantial effect on the survival of *Candida albicans* 488.

## 3. Materials and Methods

### 3.1. Materials

**Figure 12:**
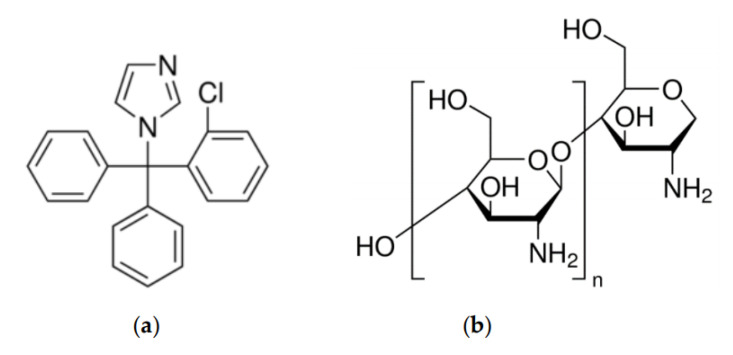
Chemical structure of: (**a**) clotrimazole, and (**b**) chitosan repeated monomer unit.

Clotrimazole (CL) used in the study (Figure 12a) was obtained from Hasco Lek S.A. (Wrocław, Poland). Sodium lauryl sulphate (SLS) was purchased from P.P.H. Stanlab (Lublin, Poland). Ethanol was obtained from Chempur (Piekary Śląskie, Poland). High-molecular weight chitosan (CHS) (Figure 12b), with 92% deacetylation degree and a viscosity-average molecular weight of Mv =1087 kDa, was purchased from France-Chitine (Orange, France). Samples of chitosan A (CHA) and chitosan B (CHB) were prepared in the Institute of Applied Radiation Chemistry Technical University of Łódź (Łódź, Poland) by irradiation of dry CHS in an air atmosphere by greys from a ^60^Co source (average energy of quantum 1.25 MeV) at 5 and 30 kGy, respectively. The average viscosimetric molecular weight of chitosan samples, presented in Table 7, was determined by the Ubbelohde capillary viscometry technique.

### 3.2. Mixture Preparation

Mixtures of clotrimazole–chitosan were prepared by grinding (GM) and kneading (KM) methods after mixing clotrimazole and the selected polymer in different ratios.

#### 3.2.1. Grinding Method (GM)

Mixtures of CL–CHS, CL–CHA, and CL–CHB were prepared by grinding mixtures of each component in an agate mortar for 10 min. The weight ratios of components in mixtures of clotrimazole–polymer were 1:9, 3:7, 5:5, 7:3, and 9:1. The obtained mixtures were sieved through a 315 µm mesh and stored in a desiccator at room temperature until use.

#### 3.2.2. Kneading Method (KM)

Clotrimazole and chitosan were weighed and mixed in an agate mortar with the addition of a sufficient volume of ethanol to achieve a slurry-like consistency. The solvent used to prepare mixtures was then completely evaporated at 30–35 °C with continuous stirring to obtain a dry mass. The dry mass was then triturated in an agate mortar and sieved through a 315 μm mesh. The pulverised solid dispersions were stored in a desiccator at room temperature until use. The weight ratios of CL–CHS, CL–CHA, and CL–CHB in the obtained mixtures were 1:9, 3:7, 5:5, 7:3, and 9:1. 

### 3.3. Determination of Drug Content

Samples of obtained mixtures containing the equivalent of 10 mg CL were dissolved in 10 mL of methanol. The solutions were filtered and diluted by methanol in a 1:10 ratio, and the clotrimazole concentration in solutions was determined spectrophotometrically at λ = 260 nm using a UV–Vis spectrophotometer (Jasco V-650, Tokyo, Japan). Clotrimazole concentrations in the mixtures were calculated against a predetermined calibration curve (*R*^2^ = 0.999) over the concentration range of 50–500 μg/mL.

### 3.4. Physicochemical Characterisation of Clotrimazole–Chitosan Mixtures

#### 3.4.1. Fourier Transform Infrared Spectroscopy (FTIR)

FTIR spectra of clotrimazole, chitosan S, A, and B, and CL–CH mixtures were obtained using a Perkin-Elmer Spectrum Two FTIR spectrometer (Perkin Elmer, Waltham, MA, U.S.A.). The spectra were collected in the range of 450 to 4000 cm^−1^, using the attenuated total reflection (ATR) sampling mode. An ATR device with a diamond crystal was used. Each FTIR spectrum was registered at room temperature in transmittance mode, by an accumulation of 32 scans with a resolution of 4 cm^−1^. After scanning of each sample, an air spectrum recording for the clean and dry ATR crystal was taken and subtracted automatically as background.

#### 3.4.2. Powder X-ray Diffraction Analysis (XRPD)

Powder X-ray diffraction patterns for mixtures containing clotrimazole and chitosan S, A, and B were measured on a powder diffractometer (D2 Phaser, Bruker, Germany) equipped with CuKα radiation and 1D Lynxeye detector. The diffraction patterns were measured in the range of 7°–50° (2*θ*) with a 0.02° step (2*θ*) and an exposure time of 0.5 s per step.

#### 3.4.3. Differential Scanning Calorimetry (DSC) 

DSC curves of pure components and CL–CH mixtures were recorded using a 214 Polyma (Netzsch, Selb, Germany) heat flux-type calorimeter. The measurements and data analysis were carried out using Proteus 7.0 software (Netzsch, Selb, Germany). Calibration of the DSC instrument was performed using the melting points of indium (156.6 °C), tin (231.9 °C), bismuth (271.4 °C), and zinc (419.5 °C) as standards [42]. DSC samples were prepared by weighing approximately 4–5 mg of CL, CH, or the appropriate CL–CH mixture in a standard 40 µL aluminium crucible. The crucible was next sealed with a pierced lid. An empty standard crucible, with a pierced lid, was used as reference. DSC measurements were run in triplicate, in the temperature range of 10–160 °C and a heating rate of 5 °C min^−1^. Dry nitrogen (99.999% purity), with a flow rate of 50 cm^3^ min^−1^, was used as a purge gas.

### 3.5. In Vitro Dissolution Release Profile of Clotrimazole

To perform dissolution studies, samples of 100 mg clotrimazole and CL–CH mixtures were pressed under a pressure of 1 tonne using a hydraulic press (Specac, Orpington, UK). In vitro drug release studies were carried out in triplicate, using the U.S.P. type 1 apparatus (Vankel VK7025, Varian Inc., Cary, NC, USA) with 500 mL of water with 1.0% SLS as dissolution medium at a paddle speed of 50 rpm and temperature of 37 ± 0.5 °C. At predetermined intervals (5, 10, 15, 20, 25, 35, 40, 50, and 60 min), 3 mL aliquots were withdrawn, and clotrimazole concentrations were determined using a UV–Vis spectrophotometer (Jasco V-650, Tokyo, Japan) at λ = 260 nm. Clotrimazole concentrations in mixtures were calculated against a predetermined calibration curve (*R*^2^ = 0.997) over the concentration range of 2–20 μg/mL.

### 3.6. Antifungal Activity

#### 3.6.1. Determination of Minimal Inhibitory Concentration (MIC) of Clotrimazole and Chitosan S

The minimal inhibitory concentrations of clotrimazole (MIC_CL_) and chitosan S (MIC_CH_) were tested in acid and neutral conditions following standard and modified EUCAST procedures [43]. The susceptibility tests were performed on 10 clinical isolates of *Candida albicans* and 7 clinical isolates of *Candida glabrata*, as well as *Candida albicans* ATCC 90028, *Candida albicans* ATCC 10231, and *Candida glabrata* ATCC 90030. The strains were stored at −80 °C and were cultured on Sabouraud Dextrose Agar and incubated at 37 °C before testing. After 24 h, the cultures were resuspended at a density of 1–5 × 105 CFU/mL and used to inoculate the 96-well microplates loaded with different dilutions of tested compounds.

##### Standard Procedure (pH 7)

In accordance with the EUCAST (European Committee on Antimicrobial Susceptibility Testing) document, the microdilution method and liquid medium RPMI 1640 (supplemented with 2% glucose and buffered with MOPS (4-Morpholinepropanesulfonic acid)) of pH 7 were used. The stock ethanol (96%) solutions of clotrimazole (1600 mg/L) were used to prepare serial ethanol dilutions ranging from 3.1 to 800 mg/L, which were subsequently diluted 100 times in double-concentrated RPMI 1640 (RPMI 1640 2x). After the addition of equal volume (50 µL) of the water solution of tested microorganisms, the final concentrations of clotrimazole were 0.0078–8 mg/L. The stock solution of chitosan (10,000 mg/L in 1% acetic acid) was used to prepare serial dilutions in 1% acetic acid to obtain concentrations of 19.5–2500 mg/L. Finally, 5 µL of each solution was transferred to 45 µL of RPMI 1640 2x dispersed in appropriate wells of the microplates. After adding 50 µL of microbial suspensions, the range of obtained chitosan concentrations was 250–0.8 mg/L.

##### Modified Procedure (pH 4)

Experiments were performed as in the standard procedure, except we used acid-RPMI 1640 (RPMI 1640 supplemented with 2% glucose, 0.1% acetic acid and MOPS) at pH 4 and prepared yeast suspensions in 0.1% water solution of acetic acid. Chitosan S was tested in the same concentrations as in the standard method, but clotrimazole concentrations were higher. The stock ethanol solution of clotrimazole was 25,000 mg/L, working solutions ranged from 50 to 12,500 mg/L, and the final tested concentrations were 0.25–125 mg/L. The plates, prepared according to standard or modified procedures, were incubated at 37 °C for 24 h and then measured spectrophotometrically (530 nm). MIC was calculated as the lowest concentration of the tested drug that produced a 50% reduction in the strain absorbance compared to the absorbance of the negative (untreated) control.

#### 3.6.2. Determination of Minimal Inhibitory Concentrations (MIC) of Clotrimazole–Chitosan S Combinations

The MIC of clotrimazole–chitosan S combinations were assessed on three strains of *Candida glabrata* and one *Candida albicans*. In the chequerboard assay, the wells of the 96-well microplate were inoculated simultaneously with both tested compounds and the investigated microorganisms. The combinations of clotrimazole and chitosan were also tested in acid and neutral conditions using RPMI 1640 media. The range of concentrations of chitosan tested in acidic conditions was 0.25–500 mg/L, whereas clotrimazole was 0.25–125 mg/L for isolates of *Candida glabrata* and 0.0156–8 mg/L for the isolate of *Candida albicans*. In neutral conditions, the range of tested clotrimazole concentrations was 0.0156–8 mg/L, and chitosan S was 7.8–500 mg/L. After a 24-h incubation, the absorbance at 530 nm was measured. The fractional inhibitory concentration of clotrimazole (FIC_CL_) was calculated as MIC_CL-CH_ of clotrimazole obtained in the presence of chitosan, divided by MIC_CL_ of clotrimazole alone. The fractional inhibitory concentration of chitosan (FIC_CH_) was a quotient of MIC_CH-CL_ of chitosan S acting in combination with clotrimazole and MIC_CH_ of chitosan acting alone. The sum of FIC_CL_ and FIC_CH_ represents the fractional inhibitory concentration index (FICI), which is indicative for synergism (SYN) if its value is less than 0.5, indifference (IND) if ranged from 0.5 to 4, and antagonism when it is higher than 4 [44,45].

#### 3.6.3. Antifungal Activity of Clotrimazole in the Presence of Chitosan

Our experiment tested the antifungal activity of the mixtures prepared with a defined ratio of clotrimazole–chitosan, namely 1:9, 3:7, 5:5, 7:3, and 9:1. For each of the tested strains, two concentrations of clotrimazole were prepared (one equal to MIC, and a second representing ½ of MIC) and pipetted into five wells of the microplate. Next, the appropriate amounts of chitosan were added, keeping the defined ratio. After a 24-h incubation at 37 °C, the viability of the tested samples was measured at 530 nm. Results were expressed as a percentage of fungal growth inhibition compared to control wells, where the fungal growth was 100%.

## 4. Conclusions

Previous studies have investigated the impact of different types of chitosan on yeast species, including *Candida*. The results demonstrated the anti-yeast activity of high-molecular weight and low-molecular weight chitosans [46]. In this work, we analysed the physicochemical properties and activity of high-molecular weight chitosan (M.W. > 400 kDa) and clotrimazole against clinical isolates of *Candida* species. The dissolution results obtained in our work led to the conclusion that chitosan can be used as an excipient which improves the dissolution rate of poorly soluble clotrimazole (II class in the Biopharmaceutical Classification System). The dispersion of drug molecules between the polymer chains increases the effect of wetting the particles of medication and prevents their agglomeration [47,48]. We observed the fastest dissolution of the drug in mixtures containing chitosan S (which has the highest molecular weight) and proceeded to analyse their activity against both albicans and non-albicans strains. We observed significant differences related to the susceptibility of tested strains to CL–CHS. The examined CL–CHS combinations demonstrated significant activity against *Candida*, especially *C. glabrata* strains, which have an innate resistance to azole antimycotic therapy [49,50,51]. Our studies indicate that high-molecular weight chitosan and clotrimazole could have a synergistic effect on C. glabrata isolates at acidic pHs. The development of pharmaceutical formulations based on clotrimazole–chitosan solid-state mixtures is a promising step towards developing alternative effective topical antifungal formulations.

## Figures and Tables

**Table 1:** The compositions of clotrimazole–chitosan mixtures prepared by grinding (GM) and kneading (KM) methods.

	Mixture Code		Drug/Chitosan Weight Ratio
Chitosan S	Chitosan A	Chitosan B	
grinding method (GM)			
CL1-CHS9 GM	CL1-CHA9 GM	CL1-CHB9 GM	1:9
CL3-CHS7 GM	CL3-CHA7 GM	CL3-CHB7 GM	3:7
CL5-CHS5 GM	CL5-CHA5 GM	CL5-CHB5 GM	5:5
CL7-CHS3 GM	CL7-CHA3 GM	CL7-CHB3 GM	7:3
CL9-CHS1 GM	CL9-CHA1 GM	CL9-CHB1 GM	9:1
kneading method (KM)			
CL1-CHS9 KM	CL1-CHA9 KM	CL1-CHB9 KM	1:9
CL3-CHS7 KM	CL3-CHA7 KM	CL3-CHB7 KM	3:7
CL5-CHS5 KM	CL5-CHA5 KM	CL5-CHB5 KM	5:5
CL7-CHS3 KM	CL7-CHA3 KM	CL7-CHB3 KM	7:3
CL9-CHS1 KM	CL9-CHA1 KM	CL9-CHB1 KM	9:1

**Table 2:** Cumulative percentage of drug released from CL–CH mixtures prepared by grinding (GM) and kneading methods (KM) 60 min after the dissolution test.

Type of Formulation	% of Drug Release	Type of Formulation	% of Drug Release	Type of Formulation	% of Drug Release
Chitosan S		Chitosan A		Chitosan B	
**Grinding method**					
CL1-CHS9 GM	77.42 ± 1.03	CL1-CHA9 GM	62.77 ± 1.69	CL1-CHB9 GM	48.95 ± 0.99
CL3-CHS7 GM	45.77 ± 0.99	CL3-CHA7 GM	42.99 ± 0.50	CL3-CHB7 GM	39.52 ± 0.75
CL5-CHS5 GM	39.21 ± 0.64	CL5-CHA5 GM	39.14 ± 1.18	CL5-CHB5 GM	36.37 ± 1,17
CL7-CHS3 GM	30.20 ± 0.83	CL7-CHA3 GM	29.90 ± 1.73	CL7-CHB3 GM	26.98 ± 3.11
CL9-CHS1 GM	11.94 ± 1.50	CL9-CHA1 GM	16.91 ± 8.03	CL9-CHB1 GM	14.64 ± 6.82
**Kneading method**					
CL1-CH9 KM	48.99 ± 1.16	CL1-CHA9 KM	44.00 ± 1.54	CL1-CHB9 KM	43.84 ± 0.83
CL3-CH7 KM	35.34 ± 1.05	CL3-CHA7 KM	32.79 ± 1.57	CL3-CHB7 KM	30.62 ± 2.87
CL5-CH5 KM	29.84 ± 0.85	CL5-CHA5 KM	29.65 ± 1.43	CL5-CHB5 KM	28.87 ± 1.17
CL7-CH3 KM	26.64 ± 2.09	CL7-CHA3 KM	25.83 ± 1.13	CL7-CHB3 KM	21.96 ± 2.05
CL9-CH1 KM	7.11 ± 1.38	CL9-CHA1 KM	11.54 ± 0.56	CL9-CHB1 KM	11.00 ± 6.70

**Table 3:** The temperature and enthalpy values for clotrimazole melting observed on DSC thermograms during heating of CL–CHS examined formulations.

Type of Formulation	ΔH*_fusion_* [J g^−1^]	*T_peak_* [°C]
Clotrimazole	103.5	145.6
CL9-CHS1 GM	93.7	145.0
CL9-CHS1 KM	88.1	145.1
CL5-CHS5 GM	49.9	144.8
CL5-CHS5 KM	38.8	144.8
CL3-CHS7 GM	28.4	144.3
CL3-CHS7 KM	20.5	144.1
CL1-CHS9 GM	12.6	141.2
CL1-CHS9 KM	5.6	143.5

**Table 4:** The susceptibility of *Candida glabrata* and *Candida albicans* strains to clotrimazole and chitosan S, determined in standard (pH 7) and modified conditions (pH 4).

Species	Strain Number	MIC_CL_ [mg/L]		MIC_CH_ [mg/L]	
		pH 7	pH 4	pH 7	pH 4
*C. glabrata*	ATCC 90030	0.125	3.9	>250	3.9
	769	4	31.2	>250	3.9
	773	2	15.6	>250	>250
	1941	2	31.2	>250	3.9
	2342	4	31.2	>250	>250
	2586	2	31.2	>250	1.95
	2853	1	15.6	>250	>250
	2738	1	31.2	>250	>250
	*Mean* ± SD *for 8 strains*	2 ± 2.01 ^(1)^	22.8 ± 10.02 ^(1)^	>250 ^(2)^	126.70 ± 123.20 ^(2)^
	*Median*	2.00	31.20	>250	126.95
*C. albicans*	ATCC 90028	<0.0156	0.03	>250	7.8
	ATCC 10231	<0.0156	0.06	>250	15.6
	112	<0.0078	0.06	>250	>250
	144	<0.0078	0.0156	>250	>250
	177	<0.0078	0.0156	>250	>250
	257	<0.0078	0.0156	>250	>250
	259	<0.0078	0.0156	>250	>250
	488	0.06	1	>250	>250
	1050	2	0.03	>250	>250
	1342	2	0.0156	>250	>250
	1444	<0.0156	0.0156	>250	0.8
	3089	0.06	0.25	>250	>250
	*Mean* ± SD *for 12 strains*	0.35 ± 0.74 ^(3)^	0.13 ± 0.27 ^(3)^	>250 ^(4)^	189.51 ± 104.80 ^(4)^
	*Median*	0.0156	0.0228	>250	>250

^(1)^*p* = 0.000418 (*p* < 0.001) [MIC of clotrimazole for *C. glabrata* measured in pH 7 and pH 4]. ^(2)^ *p* = 0.033147 (*p* < 0.05) [MIC of chitosan for *C. glabrata* measured in pH 7 and pH 4]. ^(3)^ *p* = 0.387469 (*p* > 0.05) [MIC of clotrimazole for *C. albicans* measured in pH 7 and pH 4]. ^(4)^ *p* = 0.081975 (*p* > 0.05) [MIC of chitosan for *C. albicans* measured in pH 7 and pH 4].

**Table 5:** Antifungal activity of the combination of clotrimazole and chitosan S tested in standard conditions (pH 7).

Strain	Clotrimazole			Chitosan S			FICI (FIC_CL_+FIC_CH_)
	MIC_CL_	MIC_CL-CH_	FIC_CL_ (MIC_CL-CH_ /MIC_CL_)	MIC_CH_	MIC_CH-CL_	FIC_CH_ (MIC_CH-CL_ /MIC_CH_)	
	[mg/L]	[mg/L]		[mg/L]	[mg/L]		
*C. glabrata 769*	4	2	0.5	>500	<7.8	0.0156	0.5156 (IND)
*C. glabrata 773*	2	2	1	>500	<7.8	0.0156	1.0156 (IND)
*C.glabrata 1941*	2	2	1	>500	<7.8	0.0156	1.0156 (IND)
*C.albicans 488*	0.06	0.06	1	>500	<7.8	0.0156	1.0156 (IND)

SYN—synergism, IND—indifference.

**Table 6:** Antifungal activity of the combination of clotrimazole and chitosan S tested in modified conditions (pH 4).

Strain	Clotrimazole			Chitosan S			FICI (FIC_CL_+FIC_CH_)
	MIC_CL_	MIC_CL-CH_	FIC_CL_ (MIC_CL-CH_ /MIC_CL_)	MIC_CH_	MIC_CH-CL_	FIC_CH_ (MIC_CH-CL_ /MIC_CH_)	
	[mg/L]	[mg/L]		[mg/L]	[mg/L]		
*C.glabrata 769*	31.25	<0.25	0.008	3.9	<0.25	0.0640	0.0720 (SYN)
*C.glabrata 773*	15.60	1	0.064	>500	<7.8	0.0156	0.0796 (SYN)
*C.glabrata 1941*	31.25	<0.25	0.008	3.9	1	0.2564	0.2644 (SYN)
*C.albicans 488*	1	1	1	>500	<7.8	1.0156	1.0312 (IND)

SYN—synergism, IND—indifference.

**Table 7:** Physicochemical parameters of investigated chitosan samples.

Chitosan Sample	Intrinsic Viscosity (η) ^1^ [dm^3^ /g]	Viscosity-Average Molecular Weight (Mv) ^2^ [kDa]
chitosan S	0.7437	1087
chitosan A	0.5843	839
chitosan B	0.2986	407

^1^ Intrinsic viscosity—molecular weight relationship for chitosan determined in 0.25 M acetic acid/0.25 M sodium acetate; ^2^ viscosity-average molecular weight (Mv), calculated from intrinsic viscosity (η) using the Mark–Houwink–Sakurada equation.
